# Risk factors and pathological characteristics for intraductal tumor spread of submucosal gland in early esophageal squamous cell neoplasia

**DOI:** 10.1038/s41598-020-62668-7

**Published:** 2020-04-22

**Authors:** Wen-Lun Wang, I-Wei Chang, Ming-Hung Hsu, Tzu-Haw Chen, Chao-Ming Tseng, Cheng-Hao Tseng, Chi-Ming Tai, Hsiu-Po Wang, Ching-Tai Lee

**Affiliations:** 1 School of Medicine, College of Medicine, I-Shou University, Kaohsiung, Taiwan; 2 Department of Internal Medicine, E-Da Hospital/I-Shou University, Kaohsiung, Taiwan; 3 Department of Pathology, School of Medicine, College of Medicine, Taipei Medical University, Taipei, Taiwan; 4 Departments of Pathology and Laboratory Medicine, Wan Fang Hospital, Taipei Medical University, Taipei, Taiwan; 5 Department of Pathology, Taipei Medical University Hospital, Taipei, Taiwan; 6 Department of Internal Medicine, National Taiwan University Hospital, Taipei, Taiwan

**Keywords:** Gastrointestinal cancer, Oesophagogastroscopy

## Abstract

The esophageal gland duct may serve as a pathway for the spread of early esophageal squamous cell neoplasia (ESCN) to a deeper layer. Deep intraductal tumor spreading cannot be completely eradicated by ablation therapy. However, the risk factors of ductal involvement (DI) in patients with ESCNs have yet to be investigated. We consecutively enrolled 160 early ESCNs, which were treated with endoscopic submucosal dissection. The resected specimens were reviewed for the number, morphology, resected margin, distribution and extension level of DI, which were then correlated to clinical factors. A total of 317 DIs (median:3, range 1–40 per-lesion) in 61 lesions (38.1%) were identified. Of these lesions, 14 have DIs maximally extended to the level of lamina propria mucosa, 17 to muscularis mucosae, and 30 to the submucosa. Multivariate logistic regression analysis showed that tumors located in the upper esophagus (OR = 2.93, 95% CI, 1.02–8.42), large tumor circumferential extension (OR = 5.39, 95% CI, 1.06–27.47), deep tumor invasion depth (OR = 4.12, 95% CI, 1.81–9.33) and numerous Lugol-voiding lesions in background esophageal mucosa (OR = 2.65, 95% CI, 1.10–6.37) were risk factors for DI. The maximally extended level of ducts involved were significantly correlated with the cancer invasion depth (*P* < 0.05). Notably, 245 (77%) of the involved ducts were located at the central-trisection of the lesions, and 52% of them (165/317) revealed dilatation of esophageal glandular ducts. Five (1.6%) of the involved ducts revealed cancer cell invasion through the glandular structures. In conclusion, DI is not uncommon in early ESCN and may be a major limitation of endoscopic ablation therapy.

## Introduction

Esophageal cancer is the eighth leading cause of death from cancer globally. Its incidence is rapidly increasing, however survival is still very poor^1,2^. An early diagnosis and prompt treatment are of paramount importance to improve survival and decrease disease burden. Endoscopic submucosal dissection (ESD) has become the standard curative treatment for early esophageal squamous cell neoplasia (ESCN), including superficial esophageal squamous cell carcinoma (ESCC) and high-grade intraepithelial neoplasia (HGIN)^3–6^. Several studies from Japan have reported good efficacy with ESD in long-term treatment outcomes^4–6^. Recently, endoscopic ablation modalities, such as radiofrequency ablation (RFA), or cryoballoon ablation, are both rapidly evolving, and some studies have shown the good efficacy and safety in treating the early ESCNs^7–10^. However, a higher recurrent risk (~20%) after ablation therapy has been reported^11^.

The submucosal glands of esophagus are generally scattered all over esophagus. These glands secrete acid mucin via the esophageal ducts which are lined by a single layer of cuboidal epithelium and open into the esophageal lumen^12^. In cases with early ESCN, the ducts of submucosal gland may serve as a path for tumor to spread to a deeper layer, so-called ductal involvement (DI)^11–13^. Because the maximal ablation depth of RFA in esophagus is the muscularis mucosae layer^14^, the deep intraductal tumor spreading could not be completely eradicated, which may potentially lead to tumor recurrence or even buried cancer. However, the risk factors and pathological features of DI in patients with early ESCNs has not been reported. Therefore, this study aimed to investigate the clinical predictors and pathological characteristics of DI with the aim of guiding management and choosing the most appropriate endoscopic modality.

## Methods

### Patients and design

From July 2008 to December 2015, we consecutively enrolled patients with early ESCN, including HGIN and superficial ESCC, who received ESD at E-Da Hospital. A complete medical history was obtained before the endoscopic treatment, including demographic and clinical data. Thorough endoscopic examinations using narrow band imaging and Lugol chromoendoscopy (1.5%) were performed for each patient to define the lesions^15^. Based on the number and multiform pattern of Lugol-voiding lesions (LVLs) in the background esophageal mucosa, the patients were initially classified into four groups^16^: (A) no LVLs; (B) several (≤10) small LVLs; (C) many (>10) small LVLs; and (D) many (>10) irregular-shaped multiform LVLs. All patients underwent endoscopic ultrasound (EUS) and computed tomography (CT) to confirm that there was no lymph node or distant metastasis. Positron emission tomography (PET) was arranged in cases with biopsy-confirmed squamous cancers or those with HGIN but questionable regional lymph node detected by EUS or CT. Informed consent was obtained from all of the patients. The study protocol was approved by the Institutional Review Board of E-Da Hospital and conformed to the Declaration of Helsinki and Good Clinical Practice guidelines.

### Endoscopic submucosal dissection

The ESD procedure was performed as described in our previous report^3^. Briefly, a circumferential incision was made initially, followed by a submucosal dissection with an IT-knife, IT-knife 2, or IT-knife nano (Olympus Co. Ltd., Tokyo, Japan). After or during the ESD procedure, adverse events including massive bleeding, emphysema, perforation and stricture were recorded. One-piece resection was defined as *en bloc* resection. R0 resection was considered to have a tumor-free margin when vertical and horizontal margins were free of tumor cells.

### Histopathological examinations of the resected specimens

**Figure 1:**
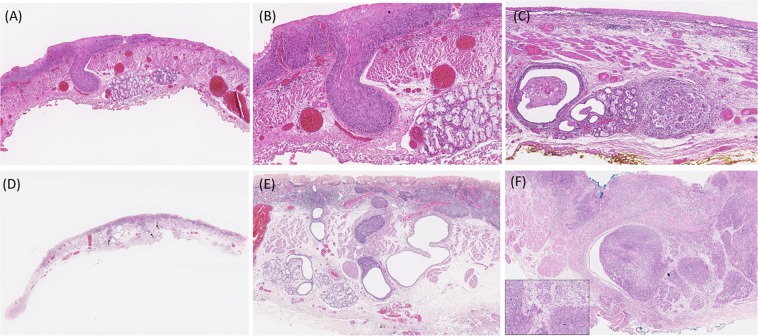
Histopathological examinations and characteristics of esophageal glandular ductal involvement (DI). (**A**) A case of intra-mucosal cancer with DI extending to the level of the submucosal layer (40x magnification); (**B**) The DI revealed the presence of ductal cancerization adjacent to normal submucosal glands (100x magnification); (**C**) A representative case of high-grade intraepithelial neoplasia with DI to the submucosal gland; (**D**) A case with many involved ducts. The slide of the specimen was divided into trisections. Of note, most of the involved ducts were located at the central part (arrow); (**E**) The DI revealed the presence of ductal cancerization accompanied by non-neoplastic ductal epithelium lined by a single layer of cuboidal cells and associated with subsequent esophageal ductal dilatation; (**F**) A case of DI in which the cancer cells invaded through the ductal structure (arrow). The area of invasion in higher magnification was shown in the inset.

The resected specimens were fixed in formalin, cut into 2-mm slices and stained with hematoxylin and eosin. The tumor size, depth of invasion, resection margins, lymphovascular invasion were histopathologically examined. The depth of invasion was subclassified as HGIN/intraepithelial cancer (m1), cancer invading the lamina propria (m2), muscularis mucosae (m3), superficial portion of the submucosa (≦200 μm, sm1), and deep submucosa (>sm1). Furthermore, two experienced doctors who were blinded to clinical history, retrospectively reviewed the presence of esophageal glandular DI (intraductal tumor spread), which was defined as the presence of ductal cancerization accompanied by non-neoplastic ductal epithelium lined by a single layer of cuboidal cells^12,13^, or complete replacement of normal ductal epithelium by carcinomatous squamous epithelial cells with the lumen filled with carcinomatous cells adjacent to normal submucosal glands and/or their ducts **(**Fig. 1). The number, morphology, margin and the maximal extension level in depth of the involved ducts were assessed. To evaluate the distribution of the involved ducts in the whole lesion, we further divided the specimens into “trisections” (Fig. 1D), and the locations of all involved ducts were recorded.

### Statistics

Statistical analyses were performed using SPSS software (SPSS for Windows, version 18.0; SPSS Inc., Chicago, IL). Comparisons between groups (DI-positive *vs*. DI-negative) were carried out using the χ2 test, Fisher’s exact test, or t-test, as appropriate. A logistic regression model was used to analyze the predictors of the presence of DI. Correlations between values were evaluated using non-parametric Spearman’s rank correlation. Significance was set at a *P* value of less than 0.05.

## Results

### Clinical characteristics of the patients

A total of 160 early-stage esophageal squamous neoplasias were treated with ESD. After the ESD procedures, 156 (97.5%) achieved en-bloc resection, and 153 had R0 resection (95.6%). Four patients had major procedure-related complications, including two with perforations and two with major bleeding and failed endoscopic hemostasis. All of whom received further esophagectomy and were discharged without sequela. Twenty-five patients developed post-ESD esophageal stenosis, and required balloon-dilatation to resolve the symptoms (median: 5, range: 1–80 sessions).

### Histopathological assessment of the post-ESD specimens

The pathological analysis of the 160 resected specimens showed that 71 (44%) lesions were HGIN and 89 (56%) were SCC (43 reaching the depths of lamina propria mucosa, 19 to the muscularis mucosae, 22 to superficial submucosa and 5 to deep submucosa). Ten lesions (6%) with lymphovascular invasion (LVI) developed in the patients with SCC (4 m3, 2 sm1 and 4 > sm1). Sixty-one (38.1%) lesions had esophageal glandular DI, of which 14 maximally extended to the level of the lamina propria mucosa, 17 to the muscularis mucosae, and 30 to the submucosal layer (Fig. 1B,C).

### Clinical predictors for the presence of esophageal DIs

The demographic and endoscopic characteristics of the patients with or without esophageal glandular DI are shown in Table 1. In univariate analysis, lesions with DI tended to be located in the upper esophagus, have invasive histology, larger tumor size, nodular surface, and larger circumferential extension. Multivariate logistic regression analysis showed that tumors located in the upper esophagus (OR = 2.93, 95% CI, 1.02–8.42), large tumor circumferential extension (OR = 5.39, 95% CI, 1.06–27.47), pre-treatment histology with SCC (OR = 4.12, 95% CI, 1.81–9.33) and numerous LVLs in background esophageal mucosa (OR = 2.65, 95% CI, 1.10–6.37) were predictors of esophageal DI (Table 2). Of note, among the patients with total circumferential lesions (n = 9), all had DI extending to the muscularis mucosae (n = 3) or submucosal layer (n = 6).

**Table 1:** The demographic and endoscopic characteristics of the patients with or without ductal involvement.

	DI-positive (n = 61)	DI-negative (n = 99)	*p-value*
Age, mean ± SD, years	52.4 ± 9.0	53.8 ± 9.8	0.36
**Gender**			
Female	0	5	0.16
Male	61	94	
BMI, mean ± SD, kg/m^2^	21.6 ± 3.4	22.1 ± 2.9	0.33
Alcohol	57	88	0.41
Betel nut	36	59	0.94
Cigarette smoking	58	87	0.17
Multiple ESCNs	24	27	0.11
**Location**			
Cervical	6	1	**0.010**
Upper	10	7	
Middle	31	63	
Lower	14	28	
**Morphology**			
Flat, type 0-IIb (n = 118)	44	74	0.716
Elevated, type 0-IIa (n = 22)	14	8	**0.010**
Depressed, type 0-IIc (n = 24)	6	18	0.177
Tumor length, mean ± SD,cm (range)	3.4 ± 1.7	2.6 ± 1.2	**0.001**
	(2–8)	(1–8)	
**Circumferential extension**			
<1/2	29	75	**<0.001**
1/2≤	18	21	
3/4≤	5	3	
Total circumference	9	0	
**Histology**			
HGIN	15	56	**<0.001**
SCC	46	43	
**Lugol background**			
Type A, B (few LVLs)	13	33	0.10
Type C, D (numerous LVLs)	48	66	

BMI, Body mass index; ESCN, Esophageal squamous cell neoplasia; HGIN, High grade intraepithelial neoplasia; SCC, Squamous cell neoplasia; LVL, Lugol-voiding lesion.

**Table 2:** Multivariate logistic regression model to predict ductal involvement.

Factors	Variables	Case No.	Odds ratio	95% CI	*p-value*
Tumor location*	Cervical-Upper *vs*. Middle-Lower	24/136	2.93	1.02–8.42	0.046
Tumor length	Mean 2.9 cm		1.15	0.85–1.57	0.366
Morphology	Nodule *vs*. non-nodule	22/138	1.04	0.34–3.16	0.947
Circumferential extension	≧3/4 vs. <3/4	17/143	5.39	1.06–27.47	0.043
Histology	SCC vs. HGIN	89/71	4.12	1.81–9.33	0.001
Lugol background^#^	Numerous vs. few LVLs	114/46	2.65	1.10–6.37	0.030

^*^Cervical-Upper esophagus indicates the location from 15 cm to 25 cm below the incisor; Middle-Lower esophagus means the location from 25 cm to 40 cm below the incisor.

^**#**^The pattern of small LVLs in the esophageal background mucosa.

### Characteristics of esophageal glandular DI

The relationships between tumor invasion depth and deepest extension level of esophageal glandular DI are shown in Table 3. Of note, among the 71 HGINs, 15 (21.1%) had DI and 11 (15.5%) extended deeper than the muscularis mucosae layer. The deepest extension level of DI was significantly correlated with the depth of cancer invasion (Spearman’s r = 0.373, *P* < 0.001).

**Table 3:** The relationship between tumor invasion depth and the deepest level of esophageal glandular ductal involvement.

Invasion depth	The deepest level of glandular ductal involvement			
	No	LPM	MM	SM
HGIN (n = 71)	56 (79%)	4 (5.6%)	7 (9.8%)	4 (5.6%)
LPM (n = 43)	24 (56%)	7 (16%)	3 (7.0%)	9 (21%)
MM (n = 19)	8 (42%)	2 (11%)	4 (21%)	5 (26%)
SM (n = 27)	11 (41%)	1 (4%)	3 (11%)	12 (44%)

HGIN, High-grade intraepithelial neoplasia; LPM, Lamina propria mucosae; MM, Muscularis mucosae; SM, Submucosa.

**Figure 2:**
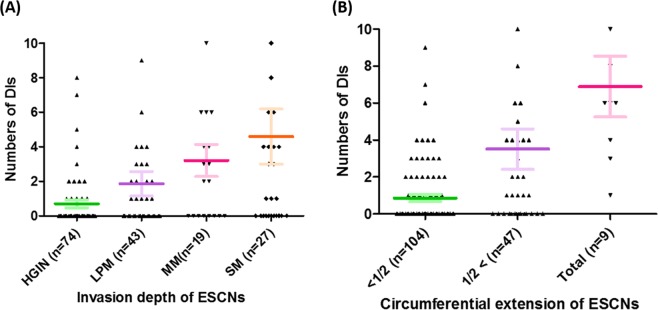
Associations between the number of involved ducts and tumor invasion depth (**A**) or circumferential extension of the tumor (**B**).

Among the 61 ESCNs with DI, a total of 317 ducts (median: 3, range: 1–40 per lesion) were identified by a detailed histopathological review. Forty-eight (79%) neoplasias had more than one involved duct. The number of involved ducts was correlated with the cancer invasion depth (Spearman’s r = 0.314, *P* = 0.014) and tumor circumferential extension (Spearman’s r = 0.353, *P* = 0.005) (Fig. 2**)**. Of note, 245 (77%) of the involved ducts were located at the central-trisection of the lesions (Fig. 1D), and 52% of them (165/317) revealed dilatation of esophageal glandular ducts (Fig. 1E). Five (1.6%) of the involved ducts revealed cancer cell invasion through the glandular structures (Fig. 1F). Three lesions showed that the resected margins were not free of involvement.

## Discussion

Endoscopic therapies, including the endoscopic resection or endoscopic ablation, had gradually become the standard treatment for early ESCNs. However, whether the presence of DIs may influence the decision making in choosing treatment modalities needs to be investigated. In this study, we found that DI was not uncommon in the patients with early ESCN (38.1%), and of these patients 79% had more than one involved duct. ESCNs located in the upper esophagus, those with larger tumor circumferential extension, tumor invasion depth and Lugol-staining patterns were associated with the presence of esophageal DI. To the best of our knowledge, this study is the first to clarify the clinical predictors of DI in early ESCNs. The clinical implication of these findings is that they may guide treatment decision making and the surveillance program.

Intraductal spread is hypothesized as a path for tumor spread to a deeper layer^11–13^. However, most endoscopists have not paid a great deal of attention to the phenomenon, and only one previous study involving 201 surgically resected superficial ESCCs evaluated the clinical significance of DI^13^. In that study, among 83 lesions with mucosal carcinomas, DI occurred in 11 (13.8%), and six (7.2%) had DI extending to the submucosal layer. These involved ducts always remained *in situ*, and the carcinoma did not spread via stromal invasion, nor was there any lymph node metastasis or impact on survival. Subsequently, in that study, the authors concluded that DI is of little significance in patients with ESCC who receive surgery. However, now that endoscopic treatment is widely applied, DI may carry significant implications on the outcomes of patients treated endoscopically. In the current study, we found that the risk of cancer cell invasion through ductal structures was very low (1.6%). When choosing ESD as initial treatment, if the esophageal glands were not totally resected, it would be difficult to evaluate whether the margin of DI was tumor free. Thus, to decrease rates of incomplete resection and recurrence, clinicians should strive to remove the esophageal submucosal glands, which appear as small white structures detected during endoscopic submucosal dissection. In addition, we found the tumors with deeper invasion depth or larger circumferential extension tend to have more DIs. Whether these neoplastic cells have higher invasion or motility ability may require further *in vitro* studies.

DI may guide the choice of endoscopic therapy for early ESCN, especially when choosing tissue-destructive treatment. Endoscopic radiofrequency ablation (RFA) or Cryoballoon ablation are both rapidly evolving treatment modalities, and some studies have shown good efficacy in treating the HGIN^7–10^. However, we recently reported the long-term outcome and found a high recurrence rate (20%) after RFA therapy^11^. While recurrent ESCNs usually presented as small and round lesions, 86% of them had DIs extension into the muscularis mucosae or submucosal layer^11^. In the current study, 15.5% of the involved ducts extended to the muscularis mucosae or submucosal layer in the lesions with HGIN. Because the maximal tumor ablation depth of RFA is the muscularis mucosae layer^14^, these deeply extended ducts will not be eradicated, which may potentially cause tumor recurrence or even buried cancer. Thus, the possibility of DI should be taken into account and may be a major limitation of ablation therapy.

Currently, endoscopic modalities such as conventional endoscopy, endoscopic ultrasound or even image-enhanced endoscopy cannot detect DI. In this study, upper esophageal neoplasias, larger tumor circumferential extension, invasive histology and numerous small Lugol-unstained patterns in esophageal background mucosa were associated with the risk of esophageal DI. This finding may guide clinical decision making with regards to endoscopic treatment and surveillance. In histological examinations, we found that many of the involved ducts were located at the center of the lesions (77%), and nearly half of the involved ducts (52%) showed dilatation of the esophageal glandular duct. These signs suggest that some endoscopic modalities such as optical coherence tomography or volumetric laser endomicroscopy^17^ may be able to detect and evaluate DI before treatment in the future.

There are several limitations to this study. First, it was conducted at a single institute. Nevertheless, we demonstrated the significant impact of DI in endoscopic treatment and highlighted the importance of histological assessments of resected specimens. Second, we reviewed the post-ESD specimens retrospectively in our cohort, thus some of the esophageal glands were potentially not completely removed. A further prospective study is required to determine whether deeper submucosal dissection with total removal of esophageal submucosal glands can reduce tumor recurrence.

## Data Availability

We declare that all the data is available.
